# Prevalence and Risk Factors for *Salmonella* spp. on Pig Carcass, Before and After Chilling, in Brazil

**DOI:** 10.3390/vetsci12090803

**Published:** 2025-08-24

**Authors:** Anna Carolina Massara Brasileiro, Mariana Avelino de Souza Santos, Cláudia Valéria Gonçalves Cordeiro de Sá, Carla Susana Rodrigues, João Paulo Amaral Haddad

**Affiliations:** 1 Department of Preventive Veterinary Medicine and Epidemiology, Veterinary College, Federal University of Minas Gerais, Belo Horizonte 31270-901, Brazil; 2 CIVG—Vasco da Gama Research Center, EUVG—Vasco da Gama University School, 3020-210 Coimbra, Portugal; 3 Department of Support and Standards, Ministry of Agriculture, Livestock and Food Supply, Secretariat of Animal and Plant Health and Inspection, Brasília 70043-900, Brazil

**Keywords:** *Salmonella*, pig carcasses, risk factors, epidemiology, food safety, Brazil

## Abstract

The Brazilian Ministry of Agriculture, Livestock and Food Supply conducted a microbiological baseline study to determine the national prevalence of *Salmonella* in pig carcasses, and characterize the contamination risk according to the abattoir size under federal inspection in 2014–2015. All establishments were size-classified according to their slaughtering capacity per day. Sample weights were defined to increase the external validity of data. Samples were collected after the random selection of two half-carcasses from 76 slaughterhouses, one at the pre-chilling stage and the other at least 12 h after chilling. A total of 1544 samples were analyzed in official laboratories using Vidas SLM and SPT systems; for positive samples, we used the ISO 6579:2014. The results revealed that 10.00% (CI 7.50–13.22) of the pre-chilling carcasses tested positive for *Salmonella* spp. The medium-sized establishments exhibited the highest prevalence (18.51%). National and international market establishments had prevalences of 17.43%, and 9.05%, respectively. For carcass samples collected after chilling, the estimated prevalence was 4.58% (CI 3.13–6.65), with the highest prevalence recorded in the medium-sized establishments (7%), and values of 12.25% and 3.5% in the NM and IM, respectively. The information and knowledge thus acquired may support the further investigation and evaluation of surveillance programs, improving food safety.

## Simple Summary

Pork is one of the most widely consumed meats in Brazil and around the world. To help make it safer for consumers, the Brazilian government carried out a national study to check how often a harmful bacterium called *Salmonella* was found in pig meat at slaughterhouses. The study looked at 76 pig slaughterhouses of different sizes across the country. Samples were taken from the surface of pig carcasses both before and after cooling. Out of 1544 samples, about 10% had *Salmonella* before cooling, while this dropped to about 4.6% after cooling. Medium-sized slaughterhouses showed the highest levels of contamination. Places that only sell meat within Brazil had more contaminated samples than those allowed to export meat internationally. These results show how the size and market type of a slaughterhouse may affect the safety of pork. This kind of information is important for improving inspection programs and helping ensure that the pork reaching people’s tables is safe to eat.

## 1. Introduction

Brazilian pork meat has a significant representation on the global market, as the country is currently the 4th largest pork producer and exporter in the world [1]. *Salmonella* has been recognized as one of the most important zoonoses, thus playing an important role in public health and economic [2]. In Brazil, data on foodborne illnesses, from 2009 to 2018, show that *Salmonella* is the second most prevalent pathogen [3]. In another Brazilian study, the estimated total average cost of human salmonellosis outbreaks attributed to products of animal origin was found to be USD 1,132,368.05, in the public health system [4]. It has been estimated that 7032 *Salmonella* infections and 62 deaths in 2018, in the United States of America (USA) [5], whereas it was the second major cause of foodborne outbreaks in the European Union (EU) and European Economic Area (EEA), with 65,967 confirmed cases, resulting in 81 deaths. Higher number of cases were observed from July to September, indicating that the disease was more frequent during the hottest months [6].

Pork meat consumption is an important cause of human salmonellosis, associated with 1% to 25% of cases in industrialized countries [7], and the pathogen has been identified in all stages of pork production. In the farm, pigs can be exposed to *Salmonella* spp. by direct contact with feces from infected pigs, from pathogens in the environment, or by consumption of contaminated feed [8]. Microbial contamination can occur during slaughter, from workers, tools, or due to cross contamination from other infected carcasses [9]. The process of cutting the carcass exposes the meat surfaces, which are highly susceptible to bacterial contamination [10]. A Brazilian quantitative microbiological risk assessment (QMRA) of pig meat consumption identified *Salmonella* spp. as the main risk associated with food safety [11]. 

Serotyping is widely used as an epidemiological method to identify subdivisions of *Salmonella* species [12]. There are more than 2600 identified *Salmonella* serovars, some of which are adapted to a specific host species, causing severe diseased conditions, e.g., *Salmonella* Typhi in humans and *Salmonella* Cholerasuis in swine. However, many serovars are not correlated to a specific host; therefore, many animals can serve as hosts without presenting any clinical signs. The importance of biosecurity measures such as pest control and the prevention of contact with wild pigs can be highlighted, as these animals are important hosts and may contribute to the dissemination of microorganisms [13,14,15,16]. In stressful situations, such as during transport, in mixing lots, and during improper animal handling, the microorganism can be eliminated with feces, serving as a potential cause of carcass contamination. *Salmonella* spp. infection can occur at different stages of the production chain; hence, a set of integrated actions is required, from farm to fork, to prevent contamination and ensure food safety [17].

One of the actions required at farm level is due to the high variation in the biological properties of *Salmonella* spp., host preferences, and environmental survival, which presents a challenge for controlling the occurrence of *Salmonella*. This also implies that different production systems may require different approaches to control the various *Salmonella* serovars [18]. These measures, when integrated with the risk mitigation strategies implemented in slaughterhouses, allow for greater effectiveness in reducing risks associated with *Salmonella*.

In order to prevent foodborne outbreaks caused by harmful pathogens, official food safety control is crucial to verify and ensure the conformity of products to the required standard and assure consumer protection [19]. In 2014 and 2015, the federal inspection service, under the Ministry of Agriculture, Livestock and Food Supply (MAPA) with the collaboration of the Center for Epidemiology, Statistics and Public Health (NEEST) of the Veterinary School of the Federal University of Minas Gerais (UFMG), conducted a national baseline survey, sampling pig carcasses before and after chilling to estimate the prevalence of *Salmonella* spp. [20]. Data from this study is expected to be used to develop standards to reduce the levels of *Salmonella* in raw pork.

This study adopted a descriptive statistic approach to determine the prevalence of *Salmonella* spp. in pig carcasses, and characterize the contamination risk according to the abattoir size, in Brazil, in 2014 and 2015.

## 2. Materials and Methods

An exploratory, nation-wide, cross-sectional, prevalence study was conducted in Brazil in order to determine the presence of *Salmonella* spp. in pig carcasses before and after chilling, from October/2014 to June/2015. During the study, 128 pig abattoirs under official sanitary inspection slaughtered more than 32 million animals. A total of 1544 pig carcass swab samples were analyzed at the Federal Laboratories of Agriculture Defense (LFDAs).

### 2.1. Sampling

The sampling strategy for the exploratory study of *Salmonella* spp. was statistically developed by the Department of Inspection of Animal Products (DIPOA), with support from members of the Scientific Advisory Committee on Microbiology of Products of Animal Origin [21].

To estimate the prevalence of *Salmonella* in pig abattoirs, a two-level sampling strategy (establishments and carcasses) was used, and sample weights were defined to increase the external validity of the data [22]. 

For the definition of the sampling plan, abattoirs under official sanitary inspection were classified according to the number of pigs slaughtered in a day, as: small (up to 200 pigs), medium (201 to 700 pigs), large (701 to 1800 pigs), and very large (1801 or more pigs). Sampling was proportional to the abattoir size, as shown in Table 1 [23].

### 2.2. Sample Collection

Samples were collected by the official veterinary inspection service employees, assuming that every day of the week and all slaughtering shifts would have the same chance of being sampled. Samples, i.e., half carcasses, were collected at random, one before chilling [24] and the other at least 12 h after chilling in the same day [25]. There was no matching of the carcasses collected before and after chilling, the use of unmatched carcass samples before and after chilling, was intentional and part of the original study design, aiming to maximize the number of carcasses assessed and provide baseline evidence for future standardization. Samples were aseptically collected by swabbing the surface of the carcass, including the belly, jowl, ham, and loin, from a sampling area of 400 cm^2^, with sterile polyurethane sponge swabs pre-moistened with buffered peptone water [20,25]. Sampled swabs were refrigerated and sent to the official laboratories to test for *Salmonella* spp.

A total of 1544 samples were collected at 76 pig abattoirs, 42 of which were involved in export, identified in this study as the international market (IM), while the remaining 34 were trading in the national market (NM), as shown in Table 2.

### 2.3. Microbiological Analysis

Immunoenzyme assays were used for presumptive *Salmonella* detection in sample analyses (AFNOR Validation BIO 12/16—09/05—VIDAS^®^ easy *Salmonella* Method (SLM) or AFNOR Validation BIO 12/32-10/11- VIDAS^®^ UP *Salmonella* (SPT) for the *Salmonella* spp.). For *Salmonella* isolation, the ISO method 6579/2002 was used, using specific O and H antisera according to the White–Kauffmann–Le Minor scheme to serological confirmation [26].

### 2.4. Statistical Analysis

Data pertaining to sample collection and laboratory results were stored in electronic spreadsheets, and the status of samples and establishments was characterized after verification and adjustments. Statistical analysis was performed using survey analysis with sample weights based on production capacity and sampling unit, as follows:

$$\mathrm{Sample}\;\mathrm{weight}:\;\frac{total\;number\;of\;slaughterhouses}{number\;of\;slaughterhouses\;sampled}\times \frac{total\;production\;of\;the\;slau}{number\;of\;samples\;of\;the\;slaughterhouse\;sampled}$$

Sample collection data and the laboratory results were analyzed using the statistical software Stata 15 (Stata Statistical Software: Release 15. College Station, TX, USA: StataCorp LP).

### 2.5. Geoprocessing

For the geoprocessing of pork slaughterhouses, all active operating facilities in 2014 and 2015 were identified, and their respective addresses and authorizations for marketing fresh meat to the domestic (NM) and foreign (IM) markets were verified. This information was obtained from the SIGSIF system and the official website of MAPA [27]. Geographic coordinates were subsequently retrieved using the Google Maps platform and incorporated into the ArcGIS Pro Version 3.5 [28] for geoprocessing.

## 3. Results

The national prevalence of *Salmonella* spp. in pig carcasses before chilling was 10.0% (95% CI 7.5–13.2). The frequency of *Salmonella* spp. in medium-sized abattoirs was 18.5% (95% CI 9.3–33.6), which was higher compared to other pig abattoirs, with a marginally significant difference (*p* = 0.051). The national prevalence of *Salmonella* spp. in pig carcasses after chilling (AC) was 4.6% (95% CI 3.1–6.6), and there was no statistically significant difference between the abattoirs, according to the number of pigs slaughtered per day (Table 3). However, difference was observed in terms of the market in which the abattoirs were involved, i.e., the prevalence of *Salmonella* spp. at the exporting abattoirs was 3.6% (95% CI 2.1–5.9), whereas that at national marketing abattoirs was 12.3% (95% CI 7.7–18.8).

The prevalence of *Salmonella* spp. at the pre-chilling stage at the national marketing abattoirs was 17.4% (95% CI 12.0–24.6), while that at the exporting abattoirs was 9.0% (95% CI 6.4–12.7).

*Salmonella* prevalence was not evenly distributed among the different regions of the country, with the highest prevalence being recorded in the Southeast, where most of the medium-sized establishments are found, followed by the South and Central-West of Brazil (Table 4).

In Brazil, the South region was responsible for 69.56% of the national pig slaughtering in 2015, followed by the Southeast (16.15%), Central-West (14.24%), and Northeast (0.05%). In the same year, 84.8% of pork production was commercialized in the NM, and 15.2% was intended for the IM [29].

During the study, 51.6% (66/128) of the pig abattoirs under federal inspection produced meat for national consumption, and the remaining 48.4% (62/128) of the abattoirs exported to 89 countries in 2014 [29] and to 72 countries in 2015 [30].

**Figure 1:**
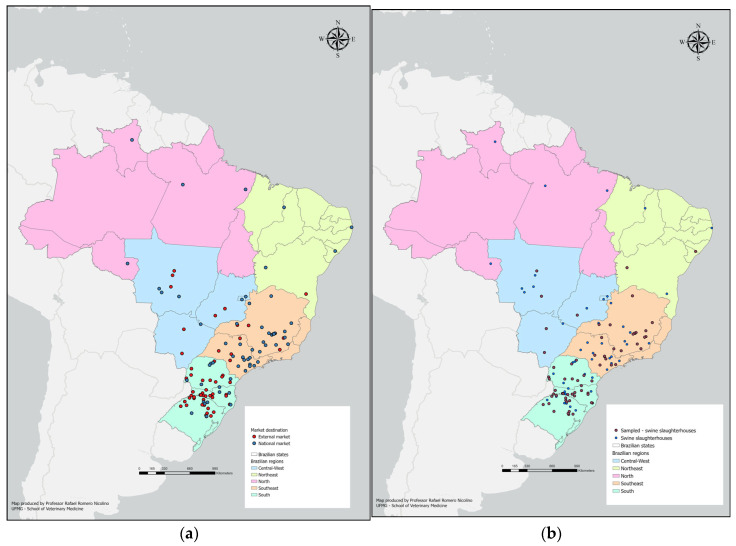
(**a**) Spatial distribution of pig abattoirs under federal inspection service according to marketing classification, Brazil, 2014–2015. (**b**) Spatial distribution of pig abattoirs that were sampled, Brazil, 2014–2015.

Figure 1a shows the distribution of Brazilian pig abattoirs under the federal inspection service, according to the marketing classification, i.e., NM and IM. The sampled abattoirs can be visualized in Figure 1b.

In addition to testing for the presence of *Salmonella* in the samples, tests were performed to identify the serotypes present in the positive samples (Table 5). It must be noted that there was no matching of carcasses collected before and after chilling.

## 4. Discussion

Brazilian results for the prevalence of *Salmonella* at the pre-chilling stage (9%) in exporting abattoirs were similar to those obtained in a *Salmonella* baseline survey conducted in the EU, from 2006 to 2007, in which 13 member states (Austria, Belgium, Cyprus, Czech Republic, Denmark, France, Ireland, Latvia, Lithuania, Poland, Slovenia, Sweden, and the United Kingdom) sampled 5736 carcass swabs before chilling. *Salmonella* prevalence was found to be 8.3% (95% CI 6.3–11.0) in this survey. Ireland exhibited the highest prevalence (20.0%), followed by Belgium (18.8%), France (17.6%) and the United Kingdom (13.5%). No carcass swabs tested positive in Latvia, Slovenia, and Sweden. The prevalence of lymph nodes was also estimated in these countries, and was found to be 9.6% (95% CI 8.2–11.1) [31]. In a EU study conducted in 2014, the *Salmonella* prevalence, estimated from 33,099 carcass samples, ranged from 0 to 17.41%. Spain and Belgium exhibited the highest prevalence (17.41% and 12.75%, respectively) [32]. A Danish study, conducted in 2015, estimated a *Salmonella* prevalence of 1.2% in carcass swabs. It must be noted that approximately 10% of human salmonellosis cases are caused by the consumption of contaminated pork meat [33].

A baseline study conducted in the USA yielded 3.05% positive *Salmonella* results in carcass samples collected post-chilling [34], similar to the results from Brazilian exporting abattoirs. Another *Salmonella* baseline study conducted in the USA on raw pork meat during 2017–2018, using 4014 samples collected from 285 establishments with different production sizes, reported a national prevalence of 28.9% (CI 24.1–33.8) in comminuted meat, 5.3% (CI 4.3–6.4) in intact meat, and 3.9% (CI 0.6–7.2) in non-intact meat [35].

The lower *Salmonella* prevalence observed after chilling might be attributed to lesser initial contamination, efficiency of carcass washing in reducing pathogen transmission, and proper chilling process and carcass disposal in the chilling chamber, allowing proper ventilation and faster cooling, resulting in unfavorable temperature and humidity conditions for *Salmonella* multiplication [36]. Carcass *Salmonella* prevalence may vary as a function of the stage in the slaughter line at which the carcass is tested; therefore, the carcass sampling point might exert an influence on the final result [37]. The microorganism can be present on the pig’s skin, in the oral cavity, feces, or lymph nodes [8], implying that cross-contamination might be enhanced during the slaughtering process [11,38], possibly due to improper implementation of sanitary operational procedures [18] or inspection lines, since during these procedures, incisions are made on the tonsils and mesenteric lymph nodes, which can become contaminated with *Salmonella*, even with the monophasic variant, *Salmonella* Typhimurium 1,4,[5],12:i:- [39].

According to [40], *Salmonella* contamination is particularly high at multiple stages of the slaughtering process in the Spanish pork production chain, including in animal transportation, holding pens, and several points of the slaughtering line, due to the high number of animals raised in different systems and regions. A Brazilian study conducted in the state of Santa Catarina yielded similar results about the higher possibility of samples testing positive for *Salmonella* in slaughterhouses, and is related to the final step that is responsible for enhancing *Salmonella* transmission, and the high number of carriers responsible for the delivery of pig batches to slaughterhouses [41]. 

The official control of *Salmonella* spp. in swine carcasses, was implemented in 2018 through the Normative Instruction SDA No. 60/2018 [42]. The monitoring protocol establishes a single sampling cycle per establishment, collected before chilling, consisting of seven samples (*n* = 7), with a maximum tolerance of one positive sample (c = 1) per cycle, with expected prevalence of 12% and probability of 80% for all abattoirs size except for medium-sized version, which has a probability of 85%. Between 2019 and 2023, the number of samples analyzed annually ranged from 559 to 589. The occurrence of *Salmonella* spp. showed slight fluctuations during this period, starting at 6.20% in 2019 (36 positive samples out of 581 analyzed), decreasing to 5.60% in 2020 (33/589), and subsequently reaching 5.90% in 2021 (33/559). In 2022, the rate declined to 5.44% (32/588), and in 2023 it reached 5.27% (31/588). In 2023, the most frequently identified serovars were *S.* Bredeney (25.8%), *S.* Typhimurium (16.1%), *S.* Infantis (12.9%), and *S.* Derby (9.7%). Other serovars included *S.* Agona, *S.* Rissen, *S.* monophasic, *S.* Adelaide, *S.* Brandenburg, *S.* Cholerasuis, *S.* Panama, and *S.* Worthington, totaling 31 isolates [43]. This historical record demonstrates relative stability in the occurrence rates of *Salmonella spp.* in swine carcasses throughout the evaluated period.

*Salmonella* spp. risk mitigation actions, such as the adoption of the all-in and all-out system, are commonly used in pig farms, due to the difficulty of risk mitigation at the end of the production chain [44]. The control of salmonellosis should be initiated in the field, through the adoption of good production practices, such as integrated pest control within farms.

The difference between the abattoirs licensed to operate in the NM and IM, in terms of the prevalence of *Salmonella*, may be attributed to the stricter sanitary requirements of establishments allowed to commercialize in the IM, improving the suppliers’ quality programs and leading to better results of *Salmonella* prevalence. The EU, USA, and States of Customs Union have specific sanitary requirements that require to be implemented and validated before trading commences. It aims at improving food safety, achieved through sequential microbiological testing of carcass samples. The performance criteria are available in the Commission Regulation (EC) Nº. 2073/2005 for Aerobic colony count, Enterobacteriaceae and *Salmonella*; Customs Union Decision (CDS) Nº. 299/2010 for Quantity of Mesophilic Aerobic and Facultative Anaerobic Microorganisms (QMAFAnM), *Colibacillus*, *Salmonella,* and *Listeria*; Title 9 of the Code of Federal Regulations (CRF) for *Salmonella* and *Escherichia coli*. These parameters are used as process hygiene indicators, aimed at improving food safety [24,45,46].

The regional variation observed may be explained by the concentration of medium-sized slaughterhouses in the Southeast, most of which are limited to commercialization within the national market and exhibited marginally higher *Salmonella* prevalence. A plausible hypothesis is that these establishments, while handling a substantial production volume to meet the high pork demand in the Southeast—the most populous region of Brazil—may operate with comparatively lower levels of technification when compared to large-scale slaughterhouses, thereby increasing the likelihood of contamination. The large and very large abattoirs are concentrated in the South, and most of them has allowance to commercialize in the IM and also NM. A study conducted in the state of Santa Catarina showed that *Salmonella* prevalence, and the variability therein, is influenced by some variables, such as the slaughtering carrier batches, quality programs, slaughter days, and region [31,47]. It can also vary depending on the number of animals slaughtered per hour, abattoir equipment condition, and animal welfare [48]. Another prevalence survey conducted in 13 abattoirs in southern Brazil found similar results for the pre-chilling stage (8.7%) as in the present study [49].

For minimizing cross-contamination during slaughtering inspections, the MAPA has approved a new model of risk-based inspection, aiming at process modernization and decreasing the possibility of cross- contamination in the inspection line [21].

The main *Salmonella enterica* isolate serovars in pork carcasses were Typhimurium/4,[5],12:i:-, Derby, Typhimurium, and Panama. According to the Brazilian government, in 2014–2015, 56 outbreaks caused by *Salmonella* spp. were reported, resulting in 2255 cases, along with none of the outbreaks were attributed to an specific serovar. None of these outbreaks were linked to the consumption of pork meat [50]. The most common *Salmonella* serovars isolated from pork carcasses in Italy were Derby (24.5%), Rissen (16.3%), Typhimurium (16.3%), Agona (10.2%), London (8.2%), and Give (6.1%) [51]. However, a baseline survey on *Salmonella* prevalence in carcasses conducted in the EU in 2014 reported *S*. Typhimurium as the most frequently recovered serovar from carcass surfaces, representing 28.3% of the *Salmonella*-positive carcasses, followed by *S*. Derby (23.6%), *S*. Typhimurim monophasic (9.94%), *S*. Infantis (8.82%), *S*. Rissen (4.88%) and *S*. Bradenburg (4.88%) [52].

The monophasic variant is found worldwide, and has been increasingly associated with *Salmonella* outbreaks in the EU [52]. This serovar is a serious cause of concern for the researchers, as it is a multidrug-resistant serovar [39,53,54]. 

Beyond the challenge of controlling *Salmonella*, antimicrobial resistance (AMR) poses a major concern due to its far-reaching public health consequences. In Brazil, this issue has been addressed through the establishment of the National Action Plan for the Prevention and Control of Antimicrobial Resistance in Animal Production in 2018, which was designed to foster awareness, surveillance, and the prudent management of antimicrobial use and resistance across multiple sectors of intervention [55,56]. A systematic review of 20 Brazilian studies (1990–2016) showed that *Salmonella* isolates from swine were most resistant to tetracycline (20.3%) and sulfonamides (17.4%), while human isolates were mainly resistant to ampicillin (19.8%) and tetracycline (17%). *S. Typhimurium* accounted for 67% of resistant strains, with numerous multidrug resistance profiles identified in both hosts. These findings highlight swine as an important reservoir of AMR *Salmonella* with implications for human health [57]. A recent USA study demonstrated the value of public genomic databases for monitoring AMR in regions with expanding sequencing capacity. The exponential increase in available genomes offers a powerful resource to track AMR gene distribution, a critical threat to human and animal health [58].

In the USA, 545 isolates were recovered from 4014 samples. The main serotypes were Anatum (13.8%), Infantis (13.0%), Johannesburg (9.0%), Derby (8.6%) and I 4,[5],12:i:- (6.0%) [35]. 

*S.* Derby is also a major cause of concern for the breeders, researchers, and authorities in the European countries, as it has been linked to non-typhoidal *Salmonella* outbreaks [51,59,60,61]. Recently in Italy a total of 757 samples were collected before chilling, and *Salmonella* prevalence was estimated to be 2.6%. S. Derby also was the most found serovar [62]. 

A Brazilian survey sampling 98 carcass surfaces before chilling and 206 carcass surfaces after chilling found a *Salmonella* prevalence of 24% in both. The serovars recovered from the isolates before chilling were *S*. Typhimurium, *S*. Panama, and *S*. Derby. In the samples collected after chilling, the observed serovars were *S*. Typhimurium, *S*. Panama, *S*. Derby, and *S*. Mbandaka [41]. Samples were collected from mesenteric and submandibular lymph nodes, feces, bleeding knives, and butchering saws from small pig abattoirs in the state of Rio de Janeiro. The serovars isolated were *S.* Typhimurium (36.4%), *S.* Abony (18.2%), *S.* Give (12.7%), *Salmonella enterica* subsp. *enterica* O:4,5 (9.1%), and *S.* Heidelberg (7.3%) [63].

Recently, [64] conducted a study in China in which 32 distinct *Salmonella* serotypes were identified among 381 strains obtained from swine. The predominant serotypes were *Salmonella* Typhimurium was the most frequently isolated (133/381, 34.90%), followed by *S*. Rissen (62/381, 16.27%) and *S*. Derby (61/381, 16.01%).

An important tool for use in the slaughter process aiming reducing *Salmonella* prevalence in carcasses is QMRA. It can indicate control points where the contamination risk is higher, allowing for mitigation measures to be placed in the slaughter line to guarantee lower carcass contamination [65,66]. Contamination and cross- contamination can occur during slaughter due to the opening or removal of oral cavity, tonsils, intestines, and pluck set. These parts can be highly contaminated, and present a risk of spreading the bacteria, since the animals are potential *Salmonella* carriers [41,65,67]. 

A comparative Brazilian study, between risk-based inspection and traditional inspection system, to investigate *Salmonella* spp. prevalence in carcass, using a total of 200 carcasses, with sample collection with the same methodology presented in this study, had 4.61% of positiveness [68].

In addition to preventive measures aimed at reducing cross-contamination during slaughter operations there are several pre-slaughter and on-farm interventions that can minimize the prevalence of this pathogen. Among these, particular emphasis should be given to animal welfare during the pre-slaughter phase, as stressed animals tend to shed the agent more intensively in their feces, as well as to the implementation of strict biosecurity practices at the farm level [67]. In a study conducted in Japan, it was found that wild rats could be potential carriers of *S*. Enteritidis and *S*. Infantis; thus, controlling their population is important to avoid cross-contamination between animals and their food. In addition to wild rats, it was suggested that birds can acquire the disease from the environment, and are natural reservoirs and important disseminators of the same [69]. A large multicountry study of 250 pig farms in Europe demonstrated that specific farm management and biosecurity practices can significantly influence the risk of *Salmonella* infection. Multivariable analyses indicated that lower-risk farms were more frequently characterized by smaller herd sizes (<400 sows), the use of rodent control measures near pig enclosures, the isolation of sick pigs, adequate downtime between farrowing batches, and fully slatted flooring in fattener units. These findings suggest that relatively simple and targeted interventions at the farm level may effectively reduce *Salmonella* transmission, reinforcing the importance of biosecurity as a complementary strategy to slaughterhouse hygiene measures. In this context, the establishment and maintenance of a secure perimeter barrier around pig farms is also crucial, not only to limit access by unauthorized personnel but also to prevent the entry of wild boars, which are recognized reservoirs and potential carriers of the pathogen [14].

The results of the prevalence study support the elaboration of the microbiological control program for pork and beef carcasses applicable to abattoirs under the federal inspection service, aimed at evaluating the hygiene process and reducing the pathogenic agents, contributing to the improvement of food safety and consumer health protection. Despite the limitation regarding the year in which the study was conducted, it is important to emphasize that, as the first exploratory investigation of its kind in Brazil—a country with vast territorial extension and global representativeness in swine production and export—this work provides a valuable baseline and serves as a reference for other developing countries. Another limitation of the study was the absence of monitoring of chilling temperatures at each slaughterhouse. However, it is important to note that, under national legislation [70], chilling processes are mandatorily standardized, and carcass deboning is only permitted once the internal temperature of the muscle mass reaches 1 °C, which ensures compliance across establishments.

## 5. Conclusions

This national baseline survey provides strategic evidence that *Salmonella* prevalence in pig carcasses was influenced by slaughterhouse size, market destination, and regional distribution, with medium-sized establishments serving the domestic market in the Southeast region representing the highest risk. The predominance of *S.* Typhimurium/4,[5],12:i:- underscores its importance as a priority target for control programs. These findings reinforce the necessity of continuous surveillance systems, stratified by slaughterhouse category, to guide science-based decision-making, resource allocation, and the implementation of sustained interventions. By supporting official veterinary services in risk-based monitoring and policy development, this study contributes directly to strengthening Brazil’s role as a global leader in pork production and export while safeguarding public health.

## Figures and Tables

**Table 1:** Sampling distribution at pig abattoirs according to their size, 2014–2015.

Size of the Abattoir	No. Pigs Slaughtered/Day	No. of Samples
Small (31)	≤200	4
Medium (25)	201–700	8
Large (27)	701–1800	12
Very Large (24)	≥1800	16

No. = number. Note: Twenty-one slaughterhouses did not have size classifications assigned due to interruptions in their activities during several periods of the year.

**Table 2:** Distribution of sampled pig abattoirs according to the market, Brazil 2014–2015.

Market	Nr. of Pig Abattoirs	Nr. of Sampled Abattoirs	Nr. of Samples Analyzed
International	62	42	1092
National	66	34	452
Total	128	76	1544

**Table 3:** Prevalence of *Salmonella* spp. on pig carcasses, before (BC) and after (AC) chilling in Brazilian abbattoirs, 2014–2015.

Abattoir Size	% of *Salmonella* BC	No. of Samples	CI (95%)	% of Salmonella AC	CI (95%)	No. of Samples
Small	5.5	57	2.1–13.6	5.9	1.8–18.1	56
Medium	18.5	146	9.3–33.6	7.0	3.9–12.2	145
Large	9.3	261	5.9–14.3	5.1	2.5–10.2	263
Very Large	8.3	281	5.5–12.3	3.6	1.9–6.7	278

No. = number; CI = confidence interval.

**Table 4:** Prevalence of *Salmonella* spp. on pig carcasses by region before chilling (BC) and after chilling (AC).

Region	No. of Abattoirs	% BC	CI (95%)	% AC	CI (95%)
Southeast	44	17.9	9.2–32.0	5.9	3.2–10.5
South	62	9.4	7.0–12.6	5.0	3.1–7.8
Central-West	13	4.0	0.9–16.6	1.1	0.2–7.8

No. = number; CI = confidence interval.

**Table 5:** *Salmonella* serovars isolated from pig carcasses, before (BC) and after (AC) chilling in Brazilian abattoirs, 2014–2015.

*Salmonella* Serovars Before Chilling	Number of Isolates	*Salmonella* Serovars After Chilling	Number of Isolates
Typhimurium/4,[5],12:i:-	11	Typhimurium/4,[5],12:i:-	10
Derby	9	Derby	6
Typhimurium	8	Typhimurium	4
Panama	4	Panama	1
Oslo	3	Infantis	1
Anatum	3	Lexigton	1
Panama/Rubislaw	2	Anatum	1
Ohio	2	Manhattan	1
Give	1	Rissen	1
Javiana	1	-	-
Infantis	1	-	-
Paratyphi B	1	-	-
Enteritidis	1	-	-

## Data Availability

The data presented in this study are available on request from the corresponding author due to official confidentiality restrictions. These data were provided by the Brazilian Ministry of Agriculture, Livestock and Food Supply (MAPA) and are subject to prior authorization for use, as detailed in the attached official approval document (Process No. 21000.048282/2020-58).

## Abbreviations

The following abbreviations are used in this manuscript:

CI	Confidence Interval
DIPOA	Department of Inspection of Animal Products
EEA	European Economic Area
EU	European Union
ISO	Internation Organization Standardization
IM	International market
LFDAs	Federal Laboratories of Agriculture Defense
MAPA	Ministry of Agriculture, Livestock and Food Supply
NEEST	Center for Epidemiology, Statistics and Public Health
NM	National market
No.	Number
QMRA	Quantitative Microbiological Risk Assessment
UFMG	Federal University of Minas Gerais
USA	United States of America
